# A Perioperative Blood Management Algorithm Aimed at Conservation of Platelets in Clinical Practice: The Role of the Anesthesiologist in Decision-Making

**DOI:** 10.7759/cureus.49986

**Published:** 2023-12-05

**Authors:** Kimberly L Skidmore, Naina Singh, Anusha Kallurkar, Hayden Cagle, Van S Smith III, Giustino Varrassi, Sahar Shekoohi, Alan D Kaye

**Affiliations:** 1 Anesthesiology, Louisiana State University Health Sciences Center, Shreveport, USA; 2 School of Medicine, Louisiana State University Health Sciences Center, Shreveport, USA; 3 Pain Medicine, Paolo Procacci Foundation, Rome, ITA

**Keywords:** blood bank shortages, surgery, coagulopathy, tranexamic acid, platelet transfusion

## Abstract

Platelet dysfunction and thrombocytopenia are associated with postoperative morbidity not only from modifiable preoperative factors but also from a lack of local patient blood management algorithms. In this regard, platelet transfusions have risen after the COVID-19 pandemic. Simultaneously, there has been a shortage of donors. It is logical, therefore, that each hospital should develop a triage tool, posting their algorithm on walls. Anesthesiologists should assist in planning a strategy to minimize blood transfusions while improving tissue oxygenation. A flowchart posted in each operating theatre may be customized per patient and hospital. Clinicians need reminders to draw a prothrombin time, fibrinogen, complete blood count every hour, and the appropriate threshold to transfuse. In summary, anesthesiologists are often unable to have a discussion with a patient until the preoperative day; thus, the onus falls on our surgical colleagues to reduce risk factors for coagulopathy or to delay surgery until after proper consultants have optimized a patient. The most important problems that an individual patient has ideally should be listed in a column where an anesthesiologist can write a timeline of key steps across a row, corresponding to each problem. If a handoff in the middle of the case is required, this handoff tool is superior to simply checking a box on an electronic medical record. In summary, in the operating suite, an anesthesiologist should emphasize the importance of a multidisciplinary approach. Continuing education, regular stakeholder meetings, and posters can assist in reinforcing algorithms in clinical practice.

## Introduction and background

Imbalance of supply and demand

Many surgical procedures have been associated with increasing blood product transfusions in recent years. This problem in part emerged because of the rise in incidence of numerous comorbidities such as cirrhosis, drug therapies associated with arterial vascular disease, and increasing use of herbal products with anticoagulant effects. In this regard, as an example, spine surgery even if it is not indicated for chronic degenerative disease, is performed regularly in the presence of major trauma, including motor vehicle accidents, falls, and gunshot or knife violence [1]. The proportion of such emergencies has increased since the COVID-19 pandemic. Many of these patients are complicated with associated co-morbidities and regularly take medications and herbal products that can contribute to higher risks of intraoperative bleeding. Furthermore, before the pandemic, nonelective spine surgeries in patients experiencing substance use disorder resulted in a length of stay of 22 days, with 20% of patients leaving against medical advice [2]. Fusions comprised 71% of these, while the next most common indications were infection (53%) or trauma (27%). Of the total, 30% had complications, the most common being hardware failure (14%). After the pandemic began, the incidence of substance abuse increased and the chance of spine surgery becoming emergent doubled to 38% [3].

Regional differences in the availability of blood donors

Availability of platelet donations has drastically decreased, partly because COVID-19 induced financial constraints upon individual donors, blood donor centers, and hospitals paying to process the blood. In the USA, each apheresis platelet unit transfused results in a charge to their insurance of approximately $1000 in 2018, i.e., twice the acquisition cost, per Kaiser Foundation News or any general internet search. Generally, platelets can be donated every week, limited by volunteer time or money. Our university hospital’s platelet daily demand of about a dozen units originates from a single nonprofit organization covering a large region, which collects about 60 units per day. In contrast, a nearby wealthier state experiences no shortage, where the American Red Cross (ARC) reports their core small group (active on social media and with phone calls from the ARC Donor Center) donates 900 units per day. In some centers in that wealthier state, and also in ours in New Orleans, $50 to $100 is offered to each platelet donor after their four-hour visit to the blood donor center. Most volunteers in America are not paid for donating platelets and are instead motivated to return often because of the sense of community and altruism [4]. Before 2003, every six whole blood donors experienced their platelets being pooled into a six-pack, which was equivalent to the only currently widely available option for adults, an “apheresis platelet unit.” This transformational policy did not occur in Europe, accounting for their relatively plentiful amount of platelet donations. The equipment required to collect and administer one unit of apheresis platelets is very expensive and can only be performed in a large blood donor center, not in a mobile van. Most importantly, on top of the cost of platelets, the cost to an individual suffering a hematoma can be a life-long disability from neurologic damage (Table 1).

**Table 1:** When critical organs are at risk We list various guidelines for discontinuation of drugs prior to surgeries with a high risk of bleeding in critical regions, such as the brain, spine, and vascular, along with the cost of antidotes. * Direct Xa inhibitors marked with* allow the effect of anticoagulation to be followed with measurement of Xa activity via blood laboratory values. ** ANDEXXA (see AstraZeneca package insert, coagulation factor Xa recombinant) administered within six hours of symptoms of stroke and within 15 hours of taking the direct Xa inhibitor drug. The dose was per package insert: 400mg IV over 15 minutes followed by an infusion of 4mg/min over two hours. Usual care included K-Centra (CSL Behring GmbH) 4-factor prothrombin complex concentrate (human). INR is the International normalized ratio derived from prothrombin time. Reference: [5,6]

Drug	Discontinue	Antidote	Average cost
Aspirin	5 to 10 days	Platelets	$1,000
		Desmopressin	$450
Cilostazol	48 hours		
Dipyridamole	48 hours		
Prasugrel	6 to 10 days	Bridge with Lovenox	
Ticlopidine	10 days		
Ticagrelor	5 to 10 days	Bridge with Lovenox	
Clopidogrel	5 to 10 days	Bridge with Tirofiban	
		Bridge with Eptifibatide	
		Bridge with Lovenox	
Abciximab	48 hours		
Eptifibatide	12 to 24 hours		
Tirofiban	12 to 24 hours		
Lovenox	24 hours	Protamine	$40
Streptokinase	48 hours		
Fondaparinux*	4 days	Factor 7a	$15,000
Coumadin	5 days	Fresh Frozen Plasma	$350
	INR less than 1.4	K-Centra 50 IU/kg	$5,000
		Vitamin K 10mg	$700
Dabigatran	5 days	Idarucizumab	$5,000
Rivaroxaban*	3 to 5 days	K-Centra 25 IU/kg	$2,500
		ANDEXXA**	$35,000
Apixaban*	3 days	ANDEXXA**	$35,000
		K-Centra 25 IU/kg	$2,500
Edoxaban*	3 to 5 days	K-Centra 25 IU/kg	$2,500

Preoperative risk stratification

The typical preoperative patient is increasingly likely to have a diagnosis of obesity defined by a body mass index over 30, with a prevalence approaching 45% in America. Obesity can cause liver cirrhosis, longer surgeries, and infections. Peripheral vascular disease also often coexists with obesity. The effects of commonly prescribed platelet inhibitors such as clopidogrel vary depending on patient genetics. During the psychological depression many experienced in the pandemic, comfort was sought in vitamin supplements, smoking, recreational drugs, over-eating, alcohol, or other forms of substance use disorder, all of which contribute to increased perioperative bleeding (Table 2).

**Table 2:** Conditions associated with bleeding and antidotes

Condition	Discontinue	Antidote	Cost
Blood urea nitrogen	Lab value < 40mg/dL	Desmopressin 4mcg/kg IV	$450
		Estrogen 20mg IV	$250
Cirrhosis	Platelet > 100,000/uL	rhThrombopoietin	$50,000
	Fibrinogen > 200mg/dL	Cryoprecipitate 10 unit	$1,300
	Fibrinogen > 200mg/dL	RiaSTAP 70mg/kg 5ml/m	$850
Valproic acid	2 weeks		
Vitamin E	2 weeks		
Ginkgo Ginseng Garlic	2 weeks		
Glanzmann	Genetic	Factor 7a 90mcg/kg	$15,000
Thrombasthenia			
COVID-19	6 weeks	Avoid Tranexamic acid	

An individual hospital should develop a triage tool, posting their algorithm on their walls specific to their needs. One physician facilitates buy-in from major clinical partners and then disseminates to end-users. Initially, our department led a quality improvement project at our institution with the primary aim of achieving a sustained increase in platelet donations in the community. Social media postings featuring people donating platelets once a week, word of mouth at the university, and posters in lounges within the hospital were considered. Later we realized the local blood donor center nonprofit has proprietary techniques and staff to drive volunteerism. Thus, we changed to a new aim. We sought to decrease the number of platelets transfused in our rural level-one trauma center. One way to prevent the need for platelet transfusion is to cancel or delay major surgery until the patient is optimized (Table 3). Another is to treat each identified disease while measuring the effects of medications that inhibit platelets (Table 1).

**Table 3:** Surgical techniques to salvage blood EBL: Estimated blood loss

Technique	Indications	Cost
Cells saver	Avoid if infected, EBL is about 2x Cellsaver transfused	$300
Hemobag	Reinfuse salvaged suctioned blood, postbypass	$3,000
Retrograde autologous prime, prebypass		$750k
Normovolemic hemodulition		$50
Topical hemostatic agents (Surgicel, Fibrillar, Arista, etc.)		$3,500

Last year, a review of over 138,000 papers was designed to formulate European practice guidelines. Two to eight weeks were required to correct anemia, including discriminating among the broad differential diagnoses. These researchers described a preference for intravenous iron if found deficient, instead of oral iron, definitely superior to last-minute simple transfusion of packed red blood cells (PRBC) that only masks the problem (Table 4) [5].

**Table 4:** Situations where laboratory values trigger drug Clinical situations such as neurosurgery or cardiac surgery may risk vital organ ischemia. Possible drugs or blood components to consider and the cost of administration are listed. INR is the International normalized ratio derived from prothrombin time.

Clinical situation	Drug	Cost
Uncontrolled trauma	Tranexamic acid 1g, 2mg/kg/hr	$5 per 1g
Platelet shortage	Keep hemoglobin above 9g/dL	$130 per packed red blood cell
Anemia chronic disease	Epoetin alfa	$350 per dose
Iron deficiency anemia	Isomaltoside 1000mg IV iron	$1000
Uncontrolled trauma	6 packed red blood cells then	$250 per unit
	6 fresh frozen plasma then	$150 per unit
	10 packs of cryoprecipitate then	$1300 per 10-pack unit bag
	1 platelet apheresis	$800 per apheresis
Every hour until stable:	Measure prothrombin time	$50
	Measure fibrinogen	$75
	Measure thromboelastogram	$600
	Measure platelet count	$50
Prothrombin time INR > 1.5	Fresh frozen plasma	$150
	Alternative: K-Centra	$2500
Fibrinogen < 200mg/dL	Cryoprecipitate 5ml/kg	$1300
	Alternative: RiaSTAP	$850
Platelets < 100,000/uL	Platelet apheresis	$800

Consensus statements, including European guidelines, typically contain waiting times and antidotes readily available for certain procedures [5-7]. We describe locally developed guidelines when facing surgery with a high estimated blood loss.

Measurement of platelet function

Traditionally, a platelet count below 100 is considered insufficient for most invasive procedures [8]. The symptoms and signs from a history and physical provide clues to bleeding diathesis. For example, a history of consumption of large amounts of alcohol, morbid obesity, or the presence of Hepatitis C may indicate cirrhosis. A history of consumption within the last two weeks of Ginger, Ginko, or Garlic supplements can precipitate bleeding from platelet dysfunction [9]. Subtle issues of bleeding at home or changes in PT, International normalized ratio (INR), PTT, fibrinogen, mean corpuscular volume, or hemoglobin preoperatively may trigger consultation with a hematologist. Platelet function is assessed as part of the thromboelastogram (TEG). Alternatively, when platelet dysfunction is associated with a medication, consultation with a pharmacist and drawing a platelet function assay (PFA) can help differentiate the effect of a drug, rather than only basing the delay of surgery upon the last date of administration. For example, after Brilinta, cardiac surgeons often delay surgery based on PFA rather than the last administration date. Reconciliation of the last dose of a particular drug is especially critical post-pandemic because fewer than half of medications are taken as prescribed and patients have fewer resources to obtain costly medications or delay doctor visits.

When a TEG is available, it is a set of laboratory values that usually require an hour and approximately $500. If a patient is heparinized, the special cartridge to neutralize heparin is often not available. Interpretation is complex; hence, TEG is rarely used consistently during the intraoperative management of patients at our institution. Similarly, a platelet count is a laboratory value introducing about an hour delay, although indicated to be redrawn every hour until stable. In massive traumas, often attention is elsewhere and therefore no platelet count may be measured for about six hours, partly due to the transfer of care to different departments and physicians. Sometimes a TEG and the oft-forgotten fibrinogen or simple PT are not available until long after various massive transfusions. In those cases, strict adherence to the 1:1:1:1 ratio imitating fresh whole blood is imperative, but often forgotten.

Anesthesia departments administer more than half of the nation’s blood supply. Post-pandemic, there has been a shortage of platelets [10]. Hence, in this quality improvement process pathway, we specifically assessed the utility of TEG measurements compared to more frequently drawn platelet counts, fibrinogen, hemoglobin, calcium, and PT, PTT.

Intraoperatively, the TEG results may require between 5 minutes and 55 minutes to return. The interpretation is complicated, but the results are of immense value in helping differentiate which of the various components to transfuse, as proven in liver transplant or spine surgery. Haemonetics provides a “TEG University” website to train clinicians, and a company representative to contact in case of confusion [11].

To best guide the management of platelet distribution to patients, it is key to describe each component that causes efficient blood clotting. The most modern method used to assess the clotting process is a TEG. This test provides specific values that grant insight into each component from blood donors or blood substitutes in the form of drugs that need to be administered to improve coagulation. The TEG result is displayed as a time versus amplitude figure. First, the R (reaction) value exhibits the time taken for the first measurable clot to form. Normal R values range from 5 minutes to 10 minutes. R times longer than 10 minutes require treatment, such as fresh frozen plasma (FFP) 250mL or K-Centra. The next reading produced by a TEG is the K (kinetics) value, or early alpha angle reduction, which measures the time taken to reach clot strength. Haemonetics brand TEG machines report this value as the first component of maximal amplitude (MA). These K values factor in the activity of fibrinogen, with a normal range of one to three minutes, or MA normal range. A value exceeding this normal range signifies the need for fibrinogen supplementation, such as cryoprecipitate 5ml/kg or RiaSTAP. The final additional key value produced by a TEG machine is the late MA measurement, primarily reading the level of platelet activity. The normal MA range is 55mm to 73mm; patients with decreased MA could be appropriate candidates for platelet transfusion or drugs that improve platelet function.

Many complex spine surgery centers continue to forego TEG related to prohibitive expenses. One nonpharmaceutical method to improve platelet function is maintaining core (not skin, which is inaccurate during surgery) temperature above 36 degrees Celsius. Core temperatures should be measured in the nasopharynx, esophagus, bladder, or rectum.

Keeping the bed in 10 degrees reverse Trendelenburg may prevent postoperative posterior ischemic optic neuritis after some spine surgeries that have the propensity to cause blindness, especially after more than two liters of blood loss. Unfortunately, reverse Trendelenburg positioning may increase the gravity-dependent bleeding at the lumbar spine surgical site. Ionized calcium and pH should be frequently measured and maintained above 1.1 and 7.2, respectively, to prevent bleeding. Glucose should be maintained between 80 and 150 via a continuous regular insulin drip intravenously to prevent infection, which can increase blood loss.

Blood components

Current medical literature for K-Centra implies correction of platelets is important before administration of K-Centra, but the price for each is equivalent. However, the only feasible solution in the presence of platelet shortages is to wait to administer precious platelets until all other factors are corrected. Recommendations during massive transfusion protocols stemming from major trauma were summarized in consensus format in 2014 by the American College of Surgeons Trauma Quality Improvement Project. Their experts advocate for frequent redrawing of hematocrit, platelet count, PT, PTT, and fibrinogen count. These laboratory values must be redrawn about every 20 minutes in a typical non-bypass liver transplant or every hour until stable in major gunshot wound-type trauma. An alternative to these is a TEG, similarly drawn into a blue top test tube, with results available from 5 minutes to 60 minutes, similar to the time before PT, PTT, and fibrinogen are available, with similar clinical effectiveness, depending upon the model of the machine. The hematocrit and platelet counts must be drawn into small purple top test tubes, with results often available in 15 minutes.

Preoperatively, the type and cross specimen needs to be drawn into a very tall pink top test tube, at least an hour before surgery. A check specimen will need to be drawn into a small purple top test tube at least 15 minutes later to avoid clerical error, the most common fatal type of transfusion reaction occurring one in 10,000 transfusions. This check specimen is asked to be repeated at many hospitals if more than three days have elapsed, in case the patient has developed new antibodies. A type and screen are chosen instead if the likelihood of transfuse is low, because it only provides the type such as A, B, O, and Rh + or - and also the screen for very rare antibodies, but requires weeks to locate a suitable donor. All of these test tubes must be signed, timed, and dated by the staff drawing them.

The blood bank will not release a room temperature, agitated Apheresis platelet unit to the operating theatre until the telephone call requesting immediate transfusion. Some hospitals spend $1,300 to maintain an agitator table in one operating room to avoid clumping for the life of platelets (five days) instead of clumping within an hour while remaining allowed at any time to return the platelets to the blood bank. The other components may be stored for eight hours in a cooler and must be returned for new substitute ice packs and cooler beyond that marked period. Once the other components are removed from the cooler, they must be transfused to the patient or disposed of within four hours.

A Pall filter is an orange square with a 40-micron filter and is good for any blood transfusion. If a Pall filter is not available, the typical Y-piece squeeze cylinder contains a 180-micron filter, adequate for any blood component. Warming devices heat the blood to 41 degrees Celsius. Pediatric patients often need small volumes, so the filter is within a small Y-piece called a “Blood administration filter” and tubing connected to a stopcock and a 20mL syringe. If hyperkalemia is a concern, washing packed red blood cells will reduce the potassium but the unit must be transfused within 24 hours, similar to the 24-hour limit after FFP is thawed.

Only a few tertiary care centers have access to alternatives to apheresis platelets. Cold-stored platelets have been implemented in some centers, such as the Mayo Clinic. They are used with success for up to two weeks [12]. An alternative is low-titer group O whole blood, even at the time of expiration, as it may provide platelet activity when only the supernatant is provided by the blood bank [13].

The American College of Surgeons recommends 1:1:1:1 ratios of packed red blood cells, fresh frozen plasma, cryoprecipitate, and platelets. This translates to one packed red blood cell of 200mL, one FFP of 200mL, 5ml/kg cryoprecipitate (a 10- or 20-pack), and an apheresis platelet 200mL unit [8]. It is underappreciated that if the hematocrit is below 30%, platelets transfused may leak from small suture holes partly due to low viscosity.

A similar pattern of loss of transfused platelets via small holes may occur if PT and fibrinogen are not corrected. Therefore, the physician should next transfuse FFP until the INR is below 1.6. If FFP is not available, then a physician can request the “Massive Transfusion Protocol” cooler that contains 4 PRBC and 4 FFP already thawed (to avoid the 45 minutes of thawing time). Finally, one must transfuse 3ml/kg to 5ml/kg (usually a 10-pack from 10 whole blood donors or about 200mL) cryoprecipitate, which can require an hour to thaw and arrive in the cooler. The literature from post-cardiopulmonary bypass surgeries demonstrates an adequate fibrinogen threshold before the administration of platelets is 200 or even 250, not just 150, or else platelets may leak from holes [14]. European guidelines recommend fibrinogen remain above 2.5mg/dL (250) during massive hemorrhage [15].

Patient blood management algorithm education

Education must be repeated often and through various methods to ensure anesthesiologists and surgeons understand the appropriate methods of replenishing platelets intraoperatively [16]. A lecture with a pre-test and post-test followed by debriefing by individual anesthesiology faculty may help achieve this goal, especially where students rapidly turnover on rotations. We elected to place an easily accessible checklist on a 3x5 laminated card on the anesthesia workstation bluebell cart. This checklist contained the phone number for the blood bank, such that the question of what components are immediately available for this patient may be addressed. It also included the phone number of ancillary staff that aid in the transportation of tubes for arterial blood gas (ABG), CBC, PT, PTT, fibrinogen, or TEG from the operating theatre to the lab. In addition to the checklist, several ABG syringes, short and tall Purple tops (for CBC or type and cross, respectively), and Blue tops (for PT, PTT, fibrinogen, and TEG) must be available. Instructions for computerized ordering and labeling of the lab sample were included. The checklist reminds our staff to correct fibrinogen to 200, INR 1.7, and Hematocrit to 30% before platelet transfusion. It reminds us to redraw a blue and purple top every hour and order ABG, CBC, PT, PTT, and fibrinogen until stable. If bleeding is too rapid to wait for lab results, then the checklist reminds us to transfuse in the 1:1:1:1 which is now the 6:6:1:1 ratio due to six packs transforming into one unit of cryo and platelets. If a TEG is chosen, then the phone number of an expert to interpret findings is also on the card.

Supervision of junior staff must occur frequently by staff informed of the hospital-wide “patient blood management” algorithm [16]. This algorithm should include a flowchart and numerical steps guiding the frequency of lab draws of specific tests, how to call for help, and the physician in charge of triage at the blood bank when a shortage develops [10]. A meeting with the blood bank director reveals the structure of the committee utilized for triage [16]. An anesthesiology attending is an important guide to educate the team about specific components transfused. Complex cardiac surgery results in 30% more complications where handoffs are made to new anesthesia attendings in the middle of the case [17]. For example, if supervising multiple operations, platelets may be administered too little or too late, without regard for adjuvants.

## Review

Common medications to treat coagulation-related bleeding

Epoetin Alpha 

Epopoetin is often injected subcutaneously three times a week by chronic renal failure patients to obviate frequent PRBC transfusions at a similar cost of about $200 per PRBC. Prospective studies of orthopedic patients prove anemic patients treated with iron and epoetin boost their hematocrit such that all blood product transfusions including platelets are reduced [18]. If Epopoetin is begun four weeks before surgery, in combination with intravenous iron, with a target of hemoglobin 13 g/dL rather than 15 g/dL, then fewer doses of Epopoetin were required, yet transfusion incidence remained at 3% [19].

Factor 7a

Historically, recombinant factor 7a (Novo-7) was first administered to and saved the life of a solder [20]. Next, Novo-7 was used in hemophiliac patients undergoing dental extractions, although some experienced the adverse reaction of increased coronary thrombosis. The dose was 90mcg/kg, available in a powder to mix and use within the next two hours and repeat as indicated based on PT INR. Novo-7 has been used after cardiopulmonary bypass for the last decade because it avoids intravascular volume overload and inflammatory responses to foreign transfusions. Off-label use is recommended after usual measures fail during massive hemorrhage [8]. However, thrombotic complications may outweigh the benefit even in pediatric cardiac surgery [21].

Tranexamic Acid 

Prior to the availability of Novo-7, amino-caproic acid was the mainstay during cardiopulmonary bypass, and its use then spread to multi-level spine surgery [22]. It was not very effective and brought some concerns about renal failure. Tranexamic acid (TXA) has replaced amino-caproic acid, although limited by dose-related seizures. The dose of tranexamic acid has not been optimized in spine surgery, but a trial called “OPTIMIZE” in cardiac surgery published a linear correlation between reduction in red cells transfused and TXA dose, from 2mg/kg/hr to the high-end dose of 16mg/kg/hr [23]. It has been studied in a meta-analysis of 20 randomized controlled trials, which found reduced blood loss by about 300mL; just 1g load can improve surgical site visualization and PT and INR values during orthopedic surgery. Tranexamic acid, desmopressin acetate (DDAVP), and other drugs reduce blood loss in spine surgery [24]. During hemorrhage, the dose of tranexamic acid is 1g over 10 minutes, followed by another 1g over infusion over eight hours [8]. Aprotinin was effective, but now its use is limited to Europe due to some concerns about renalilure [25].

Desmopressin Acetate

After cardiopulmonary bypass, if a patient is on hemodialysis chronically with a high BUN, the platelets will be dysfunctional from uremia. In these situations, often DDAVP 16mcg in 100mL normal saline is administered over 30 minutes to improve platelet function caused by bypass or other medications [26]. If antiplatelet drugs or Von Willebrand’s Disease (present in approximately 1% of patients) are the culprits, an expert opinion article in JAMA suggested administration of 0.3mcg/kg of DDAVP [8]. When Plavix is the cause of platelet dysfunction, even as many as five apheresis transfusions may reverse only half of the coagulopathy. Therefore, the consensus statement describes using DDAVP, TXA, and Novo-7, because these all improved the PFA.

Thrombopoietin 

If cirrhosis is suspected, consider Thrombopoietin, given at least 10 days before surgery. Vitamin K 5mg intravenously a few hours before surgery, or if the situation becomes urgent, 4-factor prothrombin complex concentrate K-Centra may be administered [27]. The dose of thrombopoietin (romiplostim) was defined at 3mcg/kg per week for two weeks because a platelet count rose above 100 x 109 in 79% of patients [28]. Median platelet counts improved from 47 to 164 at the time of surgery (p < 0.0001). Blood transfusion is unfavorable in cirrhosis during ascites and fluid overload. Caution must be exercised in correcting PT INR in liver failure as it does not track coagulopathy. Instead, during surgery, a TEG should be considered if coagulopathic [29]. Naik found fewer FFP but more cryoprecipitate (and no change in platelets) were transfused if monitoring TEG.

In cirrhosis, there is enormous variability in the target of preoperative platelet counts [30]. In multilevel spine surgery, for example, regardless of the etiology of thrombocytopenia, Chow et all found in 981 patients an odds ratio of 4.88 of PRBC, FFP, or platelet transfusions if platelet count was <100 preoperatively. If between 100 and 150, the odds ratio was two. An ASA score over 3 was associated with a 2.4 higher odds of requiring transfusions [31]. If instead, Idiopathic Thrombocytopenic Purpura (ITP) is the cause, then researchers showed oral daily eltrombopag 50mg from three weeks preoperatively to one week postoperatively, or intravenous immunoglobulin 1g/kg a week before surgery may help, although thrombosis is a risk, especially major cardiac or pulmonary embolism [32].

K-Centra

Because FFP is the most common cause of transfusion-related acute lung injury (TRALI), substitutes such as K-Centra are favored [8]. K-Centra is available as a powder similar to Novo-7 and Ria-Stapp. K-Centra at 1ml/kg (or if INR is 2-4, 25 IU/kg) may be administered over about ten minutes to correct PT [33]. When coumadin is the cause, K-Centra at 60 IU/kg may be used if INR is over 6, or if the patient was taking apixaban, edoxaban, or rivaroxaban which are direct Xa inhibitors and Xa levels can be followed [8].

Ria-STAP

An oft-forgotten blood component is cryoprecipitate. Each whole blood donation generates about 15mL of cryoprecipitate. A brand-name drug to replace fibrinogen is Ria-STAP (CSL Behring) [34]. Ria-STAP is a powder reconstituted to 5ml/min to reach a 50mg/kg total dose [35].

Monitoring considerations

The anesthesiologist may place an ultrasound-guided arterial line, central line, and peripheral line with a Biopatch to reduce risks of infection or trauma to the vessel. A simple 20g antecubital peripheral cannula can be changed with the Seldinger technique to a 7 French shorter secure IV. Injection ports contain bacterial contamination in over 30% so adequate hand gel and port scrubbing with alcohol must be maintained [36-37].

Central lines often are chosen because central venous pressure guides total fluid administration, but the number is only somewhat helpful as a trend. Newer monitors perform better at estimating preload status. Hypervolemia-induced venous hypertension coagulopathy can be prevented by a central venous pressure maintained lower than 10mmHg, yet high enough to help maintain urine output. Preoperative hypertension should be treated so that wide shifts of blood pressure do not contribute to bleeding or spine or cardiac ischemia (heralded by 1mm ST depression 80 after the QRS in leads II and/or V5). Mean arterial pressure (MAP) should be kept at 65mmHg, or if neuromonitoring indicates compromise, then a target mean pressure of 85mmHg is often encouraged. Since the advent of the Edwards Flo-Trak connected to a radial arterial catheter, the cardiac output can be continuously monitored to deduce the need for vasopressors to raise vascular resistance or the pulse oximetry PPV or HPI can predict the need for fluids. Some pulse oximeters provide continuous hemoglobin measurements. Point of care ultrasound of the short axis left ventricle can allow direct tracing of the end-diastolic area to estimate preload adequate beyond 6cm/m2 BSA.

If a spine surgery or interventional pain patient complains of back pain postoperatively, for example, an emergency MRI may be warranted to rule out epidural/spinal hematoma. Re-exploration to evacuate the hematoma is usually required within six hours to prevent permanent paraplegia. Pain in the back can also indicate an epidural abscess, similarly, requiring immediate MRI scan and surgical decompression.

Continuous monitoring of MAP must continue in these cases in the intensive care unit where nurses perform neuro-checks every hour until stable then reduce to every four hours on the ward. Systolic blood pressure must be kept below 140mmHg to prevent hematoma, yet MAP must remain above 65mmHg in most patients or above 85mmHg when signs of ischemia or increased ICP are present (such as during instrumentation or pressure from surgical instruments).

Anesthesiologists are capable of placing a lumbar drain, a 17g Tuohy needle with a 19g catheter into the CSF, as a typical epidural kit contains. This kit is just 1g larger to avoid occlusion from thick CSF. During endovascular stent or open thoracic aortic aneurysm repair, or transsphenoidal pituitary tumor resection, anesthesiologists place these lumbar drains and leave them in place for about three days postoperatively. The CSF is drained at 10cmH20 level but not more than 10ml/hour to avoid herniation. During movement from a bed to a gurney or any position changes, the drain stopcock must be turned off toward the patient to avoid sudden changes in ICP or herniation.

Neuromonitoring technicians communicate via telemedicine in real time to interpret the somatosensory evoked potentials and motor-evoked potential findings with a Ph.D. and report clinical problems to the anesthesiologist and surgeon. The anesthesiologist should maintain the best practices to improve the signal by avoiding more than half a MAC of vapor anesthetic and discussing preoperatively with the technologist. Maintaining adequate cerebral and spinal blood flow by ensuring adequate MAP is essential, appreciating the shift to the right of the autoregulation curve in hypertensive patients. The anesthesiologist may also add monitoring such as near-infrared spectroscopy (NIRS) available on the Flo-Trak called “Fore-Sight” and from other vendors. A sticker similar to a pulse oximeter is placed on the right and on the left forehead to allow titration of hemoglobin, cardiac output, or other factors that relate to mixed venous oxygen saturation. If the NIRS is below 75% or drops more than 20% from baseline, sometimes a transfusion of PRBC is indicated, or drugs to increase cardiac output.

From preoperative consultation with an anesthesiologist, intraoperative algorithms of patient blood management, and finally throughout postoperative monitoring until a patient (hopefully) ambulates home, anesthesiologists assist surgeons in planning a strategy to minimize blood transfusions while improving tissue oxygenation. A flowchart posted in each operating theatre may be customized per patient and hospital. Clinicians need reminders to draw a prothrombin time, fibrinogen, complete cell count every hour, and the threshold to transfuse. Anesthesiologists are often unable to discuss with the patient until the preoperative day; therefore, the onus falls on the surgeon to reduce risk factors for coagulopathy or to delay surgery until after proper consultants have optimized a patient.

A triage tool was developed in Europe during the COVID-19 pandemic that included severe burns with elderly, cardiac arrest where etiology is irreversible, advanced cognitive impairment, advanced neuromuscular disease, metastatic disease with expected survival less than six months, advanced immunocompromise, NYHA Class III or IV heart failure or COPD with FEV1 under 25% predicted, trauma with significant brain injury, ruptured aortic bleeding, ECMO, or other indicators of mortality expected over 80% [38]. Famously, one patient may receive 200 units of blood over the course of just three days, yet still expire. It is in difficult situations such as these that ethical implications regarding blood product availability for the entire community must be considered.

The most important problems that an individual patient has must be listed, in a column the anesthesiologist can write a timeline of key steps across a row, corresponding to each problem down the first column. If a handoff in the middle of the case is required, this handoff tool including coagulation is superior to simply checking a box on an electronic medical record (Table 5). For the development of an individual patient plan, the risk must be graded as low, medium, or high for both thromboembolic complications weighed against either low, medium, or high risk of hemorrhagic complications [39].

**Table 5:** Preoperative considerations

Anesthesiologist should consider this checklist during the preoperative evaluation
Will this surgery be likely to cause more than 2 liters of blood loss? If so then do the following.
Does the type and screen show an antibody? If so, wait for the type and cross 2 units of red cells.
Does the blood bank have 6 packed red blood cells, and 6 fresh frozen plasma ready?
Does the blood bank have ready 20 units of thawed cryoprecipitate?
Does the blood bank have ready 1 apheresis platelet?
Does the operating room have a $1,200 platelet agitation table to keep platelets for five days?
Does the patient have coagulopathy by history and physical, medications, or laboratory values?
Would delay of surgery allow correction of comorbidities, medications, or laboratory values?

Bridge therapies with easily reversible IV heparin or low molecular weight heparin are good options when the risk of stroke or heart attack is high. Despite the statistically higher risk of heart attack compared to bleeding, many proceduralists discontinue Plavix and aspirin, contributing to a three-fold increase in major adverse cardiac events [39]. Plavix may cause blood loss to rise 50% without postoperative morbidity except in intracranial surgery. Consultation with cardiologists, neurologists, and hematologists is recommended, led by the anesthesiologist, who must document a detailed plan including immediate availability of platelets or antidote medications, along with a handwritten informed consent.

The World Health Organization recognized patient blood management as necessary as early as 2010, including details such as keeping ionized calcium above 1.1mmol/L and pH above 7.2 [16]. 

## Conclusions

In summary, for the operating suite, an anesthesiologist should emphasize the importance of a multidisciplinary approach. Continuing education, regular meetings with stakeholders to review protocols, and posters can all assist in reinforcing algorithms.
